# Optimal time for laparoscopic intrauterine insemination performed on ewes detected in natural heat

**DOI:** 10.1016/j.sjbs.2022.103416

**Published:** 2022-08-17

**Authors:** Nurlan Malmakov, Martin Ptacek, Filipp Georgijevic Savvulidi, Ludek Stadnik

**Affiliations:** a Department of Sheep and Goat Artificial Insemination and Sperm Cryoconservation, Scientific Research Institute of Sheep Breeding Branch, Mynbaev, Almaty Region 040622, Kazakhstan; b Department of Animal Science, Faculty of Agrobiology, Food and Natural Resources, Czech University of Life Sciences, Kamycka 129, 165 00 Prague, Suchdol, Czech Republic

**Keywords:** Artificial insemination, Thawed sperm, Lambing rate, Natural heat

## Abstract

The aim of this study was to optimize the laparoscopic intrauterine insemination (LAI) methodology by testing different time intervals between the natural heat detection and ewe insemination. Three experiments were performed in the breeding conditions of Southern Kazakhstan. Ewes (n = 243) were exposed for one hour to direct contact with the teaser rams (once a day, morning or evening). Ewes expressing behavioral symptoms of heat were detected and inseminated with the use of LAI during 210–1290 min (3.5–21.5 hrs) after heat detection. Reproductive traits of lambing rate (LR) and litter size (LS) were recorded according to the births registered at 137 to 152 days post insemination. Our statistical model showed significance only for the effects of ewe age category and the time interval from heat detection to LAI on the LR attribute. The highest LR (38.8%) was detected in ewes at 2.5–3.5 years of age. Corrected least-square means of LR indicated 18.5 hrs. as an optimal time for LAI of ewes in natural heat. In the present study, the percentage value of lambing rate obtained at 18.5 h interval was 70.7%. Therefore, our study suggested an effective methodology to spread valuable genetic information in the sheep population in the regions of extensive farming where heat cycle synchronization is not usually performed. Importantly, our study is among the first ones that follow the European strategy to eliminate the occurrence of hormones in livestock production and the environment.

## 1 Introduction

Approximately 90% of Kazakhstani fine-wool and semi-fine-wool breeding ewes were inseminated cervically during the Soviet era (Kasymov, 1977). Ewes detected in natural heat (daily identification performed with the aid of aproned teaser ram) were subsequently double-cervical-inseminated with fresh semen (2–3 and 8–10 hrs. post-detection). Currently, still many Kazakh pedigree farms of fine-wool and semi-fine-wool sheep artificially inseminate ewes using the described scheme. On the other hand, huge progress in the genetic improvement was gained in dairy cattle due to the use of artificial insemination with thawed sperm obtained from progeny tested bulls (Lohuis, 1995). Application of thawed sperm in the sheep industry is not common, as cervical insemination results in low conception rates (Salamon and Maxwell, 1995b, Naqvi et al., 1998, Robinson et al., 2011). Good results with thawed sperm were achieved when laparoscopic artificial insemination (LAI) was performed in ewes with synchronized oestrus (Salamon and Maxwell, 1995a). This approach of LAI is known as fixed-time artificial insemination (FTAI). Conventionally, the FTAI involves synchronizing ewes with the use of exogenous hormones (injection or insertion/implantation) to be artificially inseminated over a controlled and very narrow timeframe (De et al., 2015), including without the need for heat detection (Luther et al., 2007). This is the most effective method to increase the number of females inseminated in a single day, eliminating the necessity of oestrus detection (Menchaca et al., 2017). For its excellent results, fixed-time artificial insemination has become a routine method for LAI of ewes with a hormonal synchronized heat cycle (Casali et al., 2017). In many world regions of extensive sheep farming, however, the heat cycle synchronization with the aid of progestogen pessaries or CIDR is not common and used mainly for experimental purposes (Aké-Villanueva et al., 2017, Gizaw and Tegegne, 2018, Demirhan, 2019). Furthermore, the method of heat cycle synchronization with the use of exogenous hormones injection or insertion might be time-consuming and labour-intensive under permanent pasture grazing (pers. comm.). Therefore, it is important to establish and optimize the procedure of LAI in ewes detected in natural heat with thawed sperm as a method with the potential to improve sheep genetics in the regions of extensive sheep farming. This methodology could be advantageous not only for the regions of extensive sheep farming, as it follows current European strategy to eliminate the occurrence of hormones in the environment (European Commission, 2020). In Kazakhstan, several authors investigated the LAI of ewes in natural heat with thawed sperm. Jakupov obtained a lambing rate of 61.1% in 18 maiden ewes after twice-daily heat detection with subsequent LAI (20 to 30 million of motile frozen-thawed spermatozoa) 10–14 hrs. after heat (Jakupov, 1996). However, Aybazov et al. reached a very promising lambing rate (39.2–43.7%) in ewes LAI-ed with thawed sperm performed after even once-daily heat detection (Aybazov et al., 2019). Although LAI is a minimally invasive procedure, it requires veterinary expertise, implies insufflation of irritant CO_2_ into the peritoneal cavity, and is more demanding in terms of expensive equipment and labour than other methods (Sylla et al., 2021). Equipment costs can be significantly reduced as (Sathe, 2018) suggested. Sathe (2018) also highlighted risks of LAI technique. For that reason Pau et al. (2020) successfully compared LAI with their new strategy of trancervical insemination ewes submitted to surgical incision of cervical folds. There is an ethical aspect on surgical procedures in livestock that are different accros countries. Some countries have already prohibited LAI technique, starting to adapt vaginal or cervical insemiation for frozen-thawed spermatozoa insemianation. Although these approaches have been attempted to overcome the use of laparoscopy, this technique is still the default method when obtaining greater pregnancy rate is mandatory (Sylla et al., 2021).

We hypothesized that FTAI methodology might be modified to be used under traditional extensive regions when ewes in natural heat are detected using aproned teaser rams and LAI-ed with thawed sperm at a fixed time after heat detection. The aim of the current study was to establish and optimize the nonhormonal FTAI methodology under an extensive farming system with a possible use in other specific regions.

## 2 Materials and methods

### 2.1 Animals

The study was approved by the Institutional Review Board of Kazakh Research Institute of Livestock and Fodder Production (TOO “КaзHИИЖиК”), protocol code 0115PК02596, date of approval July 25, 2014.

Three experiments of the present study were performed in the breeding seasons (October 11 to November 21) of 2014, 2015, and 2017 on two sheep farms in the Djambul and Almaty regions of Kazakhstan. Flocks of Kazakh semi-fine-wooled ewes were grazed on natural pastures with botanical composition typical for Southern Kazakhstan during the day (8 a.m.–7p.m.) and kept outdoor in the fenced lots overnight. All three experiments were carried out in flocks of 600–650 ewes. A total of 243 nulliparous (15.9%) and multiparous (84.1%) ewes were inseminated. Nulliparous ewes were 1.5 to 2 years old whereas multiparous ewes were 2.5 to 6 years old.

### 2.2 Heat detection

Teaser rams, used for identification sheep in the heat, were kept separately (no visual and sensorial contact with ewes). Feeding ration of teaser rams was composed of hay (ad libitum) and concentrates supply (300–800 g per head per day). The amount of concentrates was regulated to manage the body condition score range at 3.5 to 4.0 points.

Teaser rams with prepuce covered by aprons made from sackcloth (50 × 50 cm) joined ewe flock at a ratio of 80–100 ewes per ram (Kenyon et al., 2007) once a day for one hour from 6 a.m. to 7 a.m. or from 6p.m. to 7p.m.

In the present study, the sheep was assigned “in heat” only if all the criteria of sheep in heat, defined in the chapter „Reproduction“ (SID Sheep production handbook, 1996) were fulfilled. Each ewe detected as in the heat by teaser ram was immediately separated from the flock and put in a smaller pen, which is built inside a large pen where the ewes stand at night. All these ewes were subsequently evaluated for their body condition score (only ewes at BCS 2.75–3.0 were selected), age, health status, and exterior. Ewes that meet the criteria were inseminated with frozen sperm by laparoscopy. Ewes that did not meet the criteria were excluded from the set of LAI-ed animals. However, there were usually only a few rejected ewes (2–3 heads per flock) in the present study.

### 2.3 Semen used for laparoscopic insemination

For laparoscopy insemination, frozen sperm of two Suffolk rams was kindly provided by the University of Wisconsin-Madison, USA. Sperm was frozen in 0.25 ml straws. For LAI, straws were thawed in a water bath at 37–39 °C for 20 *sec*. The motility of thawed sperm was evaluated in eight randomly chosen straws (four straws per each ram) with the use of phase-contrast microscopy (400× magnification). After thawing, each straw contained 50 × 10^6^ motile spermatozoa. To inseminate, the content of each thawed straw was placed in a Robertson pipette (Minitube, Germany). The content of each straw was split to inseminate three ewes (about 16.7 × 10^6^ motile spermatozoa per ewe), under the recommended concentration range for sheep LAI (Maxwell, 1986). The insemination was conducted within 15 min after sperm thawing (4–5 min per ewe). All the manipulations with IDs, and LAI procedure were performed in a little house, under a controlled internal temperature (+18 to +22 °C). Most of the time, three ewes were inseminated by the content of a single straw. However, a few times during the whole study period, two last ewes were LAIed by the content of a single straw.

### 2.4 Laparoscopic intrauterine insemination

The procedure was performed by the same experienced person, who followed the general recommendations given by Evans and Maxwell (1987). Briefly, the laparoscopic insemination was performed using sterile clean instruments. The 5 mm trocar and cannula were inserted into the peritoneal cavity to the left of the midline. The trocar was removed and the laparoscope was inserted in the cannula. A hole was made by a 5 mm trocar to the right of the midline. After gas inflation and uterus visualization thawed semen was injected into the lumen of both uterine horns with the aid of the Robertson pipette and its applicator (Minitube, Germany). In the present study, there were no ewes observed with infections, or other health problems, or deceased after LAI.

### 2.5 Evaluated traits

Information about time intervals from heat detection to insemination in minutes was monitored in the present study. Inseminations were carried out 210–1290 min (3.5–21.5 hrs) after heat detection. These time frame was grouped into 2 hrs. intervals for further statistical evaluation: 270 min. = 4.5 hrs.; 390 min. = 6.5 hrs.; 510 min. = 8.5 hrs.; 630 min. = 10.5 hrs.; 990 min. = 16.5 hrs.; 1110 min. = 18.5 hrs.; 1230 min. = 20.5 hrs. (particular 2 hrs. intervals are defined in Satistical analysis chapter). Unfortunately, no inseminations were performed between 691 and 930 min (11.5 and 15.5 hrs) after heat detection. Reproductive traits of lambing rate (LR; 0 = ewe that did not give birth, 100 = ewe that gave birth) and litter size (LS, number of all born lambs) were recorded according to the births registered at 137 to 152 days post insemination. Additionally, information about the date of insemination, flock, and ewe age was noticed for further statistical evaluation.

### 2.6 Statistical analysis

All analyses were performed using the Statistical Analysis System (SAS) statistical software package according to SAS/STAT User’s Guide (SAS Institute Inc. 2009). The UNIVARIATE procedure was used for the description of the base data structure. ANOVA evaluation was performed using the MIXED procedure. For this analysis, ewes were grouped into 3 categories according to their age: 1.5–2.0 years of age; 2.5–3.5 years of age, and 4.0 years and older. According to the time interval from heat detection to insemination, ewes were grouped into 7 categories: 4.5 hrs. (ewes inseminated in 210–330 min. after heat detection); 6.5 hrs. (ewes inseminated in 331–450 min. after heat detection); 8.5 hrs. (ewes inseminated in 451–570 min. after heat detection); 10.5 hrs. (ewes inseminated in 571–690 min. after heat detection); 16.5 hrs. (ewes inseminated in 931–1150 min. after heat detection); 18.5 hrs. (ewes inseminated in 1151–1170 min. after heat detection), and 20.5 hrs. (ewes inseminated in 1171–1290 min. after heat detection). Nested effect of minutes within defined time hrs. categories was used to adjust all insemination within defined time category to particular hrs. category. Categories 12.5 and 14.5 hrs. were not used as no inseminations performed between 691 and 930 min after heat detection.

Variables of lambing rate (LR) and litter size (LS) were corrected using followed ANOVA statistical model:

$$Y_{ijklm}=FYS_{i}+AGE_{j}+H_{k}+MIN_{l}\left(H\right)+e_{ijklm}$$

where: Y_ijklm_ = variable trait (LR, LS); FYS_i_ = combined randomized flock-year-seasonal effect; AGE_j_ = fixed effect of ewe age category (j = ewes at 1.5–2.0 years of age, n = 38; j = ewes at 2.5–3.5 years of age, n = 104; j = ewes at 4.0 and older, n = 101); H_k_ = fixed effect of time interval from heat detection to insemination category (k = 4.5 h, n = 44; k = 6.5 h, n = 18; k = 8.5 h, n = 33; k = 10.5 h, n = 11; k = 16.5 h, n = 76; k = 18.5 h, n = 35; k = 20.5 h, n = 26); MIN_l_ (H) = nested effect of minutes (228–1322 min) within particular hours of insemination (7 categories, see above); e_ijklm_ = residual error.

The Tukey-Kramer method was used for the evaluation of differences between least-square means (LSM) values. A P-value of < 0.05 was considered statistically significant.

## 3 Results

The observed group of ewes was 3.11 years of age on average. Ewes were inseminated on average 779.44 min (approx. 13 hrs.) after natural heat detection using aproned teaser ram. The lambing rate after insemination at this time interval reached the average value of 36.63%, with an average number of 1.25 lambs born in a litter. These data, supported by other descriptive statistics of database structure, are reported in Table 1.

**Table 1:** Simple statistics of the database structure.

	AM^1^	SD^2^	MIN^3^	MAX^4^	CV^5^
Age of ewes (years)	3.11	1.007	1.5	6.0	32.39
Time,minutes (hrs)^6^	779.44 (12.99)	351.360 (5.856)	228.00 (3.8)	1288.00 (21.466)	45.08
Lambing rate (%)	36.63	48.278	0.00	1.00	131.81
Litter size (lambs)	1.25	0.434	1.00	2.00	34.78

1 AM – arithmetic mean.

2 SD – standard deviation.

3 MIN – minimal value.

4 MAX – maximal value.

5 CV – coefficient of variation.

6 Time – time interval from heat detection to laparoscopic artificial insemination.

For estimation of the optimal time interval of LAI, reproductive traits of LR and LS were corrected about defined factors in the model. The significance of particular factors is referred in Table 2.

**Table 2:** Significance (P–values) of factors used in statistical model for lambing rate and litter size estimation.

	FYS^1^	AGE^2^	H^3^	Min (H)^4^
Lambing rate	0.165	0.009	0.004	0.524
Litter size	0.064	0.155	0.182	0.278

1 FYS – combined randomized flock-year-seasonal effect.

2 AGE – fixed effect of ewe age category.

3 H – fixed effect of time interval from heat detection to insemination in hours.

4 Min (H) – nested effect of minutes within particular hours of insemination.

This table demonstrates that LR was significantly influenced by the fixed effect of ewe age category and the time interval from heat detection to LAI. Contrary, no evidence for statistical significance was observed for other factors on LR. No factors influenced significantly LS variable as well. For that reason, only results of ewe age category and the time interval from heat detection to insemination related to LR are presented in this study.

The highest LR of 38.8% was observed in a group of ewes at 2.5–3.5 years of age. These sheep were detected for 5.5% (P < 0.05) higher LR to 1.5–2 aged ewes, and for 3.2% (P < 0.05) higher LR to 4 years and older sheep.

**Fig. 1:**
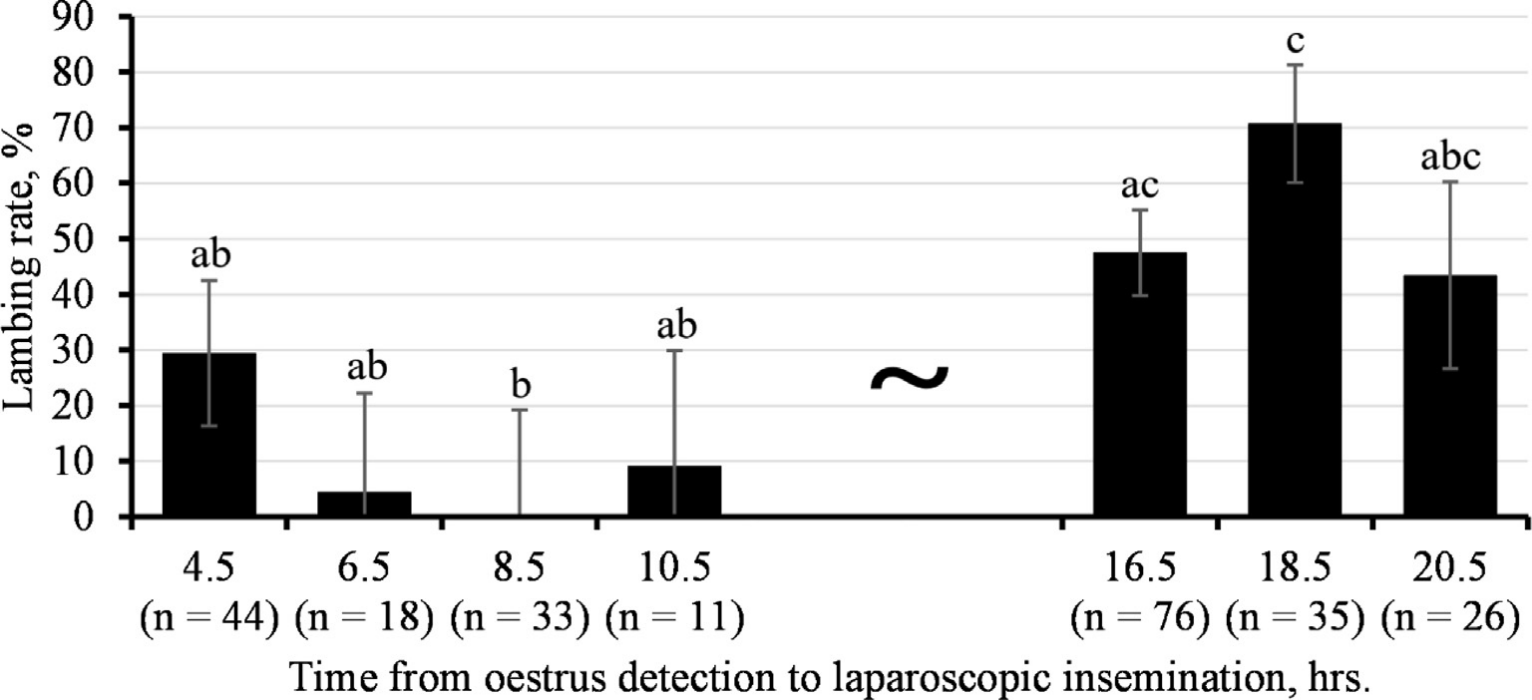
Lambing rate (LSM values ± SE) of sheep laparoscopically inseminated at time interval 4.5–20.5 h after the heat detection. a,b,c – Means within the columns with differing superscripts differ significantly at P < 0.05. LSM – least squares means. SE – standard error.

Fig. 1 represents the results of laparoscopic artificial insemination performed during the evaluated interval after natural heat detection identified using aproned teaser ram. The lowest LR (ranging from 0.00% up to 29.4%), was observed at timeframe 4.5 to 10.5 hrs. after the heat detection. On the other hand, our results indicate that the best method to obtain the highest LR is to perform LAI 18.5 h after heat detection with a teaser ram. LSM values of lambing rate reached 70.7% at this time. A significant difference between 18.5 h and all categories in the time interval 4.5–10.5 h (P < 0.05) documented this statement.

## 4 Discussion

In general, we demonstrated that the laparoscopic insemination of ewes in natural heat with thawed sperm and with a simple heat detection method is an available option to perform under very extensive conditions in a successful manner. Regarding our results, we suggested some strategies for improving LR in these conditions. Selecting animals with regard to their age should be the first tool how to perform this improvement. Animals at 2.5–3.5 years of age reached significanlty higher LR in comparison to their younger or older contenpries. These results are in full accordance with previously published those by Anel et al. (2005) or Zegeye et al. (2020). Evans and Maxwell (1987) stated that accurate knowledge of the time of ovulation is crucial to the success of insemination and that in the field conditions accurate detection of heat onset in ewes with natural heat is problematic. Twice daily heat detection allows more accurate timing of insemination relatively to ovulation (Aybazov et al., 2019, Evans and Maxwell, 1987), and it was used for intrauterine (Aybazov et al., 2019, Ham et al., 2000) or cervical (Aybazov et al., 2019, Paulenz et al., 2004) inseminations with thawed sperm of ewes in natural heat. Another approach is inducing heat cycle using a hormonal program (De et al., 2015, Casali et al., 2017, Gibbons et al., 2019, Macías et al., 2020). Methodological description of fixed-time artificial insemination (FTAI) is reviewed by Wildeus, 2000, Menchaca et al., 2017. Results of this method are well-respected and this technique is considered as a gold standard in sheep biotechnology for its effectivity and simplified methodology. However, in many world regions of extensive sheep farming, the heat cycle synchronization with the aid of hormones is not common, or not available. On the other hand, Aybazov et al. reached very promising success of LAI of thawed sperm performed after twice or even once daily natural heat detection with the use of teaser rams (Aybazov et al., 2019). This study motivated us to establish a simple nonhormonal technique of FTAI-like procedure, and to optimize the technique based on an estimation of the optimal time interval for LAI of ewes in natural heat detected by aproned teaser ram. This methodology could be advantageous not only for the regions of extensive, but also for regions of intensive sheep farming, as it follows the European strategy to eliminate the occurrence of hormones in the environment (European Commission, 2020). In this sense, biostiumulation of heat cycle should be also considered for LAI under specific breeding conditions (Rekwot et al., 2001).

As expected, LR reported in simple statistics of our study were nearly half to those performed under a precise heat detection as was reported by McCappin and Murray, 2011, Fair et al., 2005, Anel et al., 2005, or Masoudi et al. (2017). Interestingly, very similar results to ours were reached previously by Aybazov et al., who in line with our methodology investigated LR of LAI-ed ewe once-daily detected in natural heat (Aybazov et al., 2019). In agreement with Schakell et al., 1990, Cseh et al., 2012, our results confirmed that the time of insemination after heat detection impacts significantly the subsequent LR. Several cases of conceiving after the laparoscopic insemination, however, were registered outside of the defined optimal time interval were noticed in our study as well. These results confirm the previous statement of Evans and Maxwell (1987) about the frequent inaccuracy of natural heat cycle detection. As we suppose, these animals were in the optimal heat cycle phase but no properly identified in course of using a very simple method of heat detection. Being aware of the weakness of this method, we estimated very precisely the time with the highest chance to conceive and give birth to LAI-ed ewes. Corrected least-square means of LR indicated 18.5 hrs. as an optimal fixed-time for LAI of ewes in natural heat after their identification by teaser ram. Previously the optimal time for laparoscopic insemination was suggested as 12–16 h after detection of ewes in natural heat. In our study, LR at 18.5 hrs. was the highest inside the time interval 4.5–20.5 hrs., and could thus theoretically get closer to LAI performed under precise hormonal heat detection. This is a very interesting perspective for traditional flock management of extensive farming.

The technique of laparoscopic direct intrauterine insemination was developed to overcome many of the difficulties of intravaginal or intracervical insemination. The number of spermatozoa required for each insemination is lower, allowing more appropriate higher dilution rates and, therefore, better preservation protection of spermatozoa during cryopreservation. This was confirmed by Masoudi et al. (2017), who demonstrated that laparoscopic insemination was more efficient over vaginal or cervical insemination using thawed semen; however, Purdy et al. (2020) suggested perspective new non-surgical method for sheep insemination. Application of alternative methods of cervical insemination should be taken into consideration (Macías et al., 2017). Additionally, the simple method of vaginal insemination using thawed sperm that was successfully performed by Paulenz et al. (2005), and verified under UK flocks by Crilly et al. (2016) should be considered in future research as well. If the promising results are confirmed, this variation could be also implemented under organic farming in countries where hormonal stimulation of heat cycle and laparoscopic insemination are prohibited.

Currently, however, LAI is most common method for insemination with frozen thawed sperm as no other methodology gives consistently comparable or higher results. Our results show that LAI can be successfully performed under very extensive traditional sheep breeding management applied in Kazakh conditions of Central Asian steppe. Summary statistics show that the lambing rate was almost 37%. This result was achieved at approx. 13 hrs. after heat detection as obvious from basic statistics. However, our results showed that best time for LAI detected using described scheme is 18.5 hrs. Current very optimistic lambing rate can be thus even more increased. This is very perspective result how to improve LAI methodology in a very simple manner. Our results also demonstrate a possibility of introduction LAI not only to extensive condition of Central Asian steppe, but to other very traditional extensive sheep breeding conditions all over the world.

LAI was performed under Kazakh breeding conditions and all procedures followed official norms and were approved by all official conditions stated by institutional ethical comitee in studied years of realization of the expriment. Currently published studies and their new approaches must be studied and implemented very carefully to reflect requirements on this technique in all intensive- or extensive- breeding condition all over the world.

## 5 Conclusions

Our results demonstrated a perspective for using laparoscopic insemination in extensive breeding conditions without the need for hormonal heat stimulation. These results indicate that the best method to achieve the highest laming rate is to perform laparoscopic artificial insemination 18.5 h after heat detection, one time per day, with a teaser ram. LSM values of lambing rate achieved 70.7% under such scheme.

## Funding

N.M. was supported by Grant № 3788 GF4 from Ministry of Education and Science of Kazakhstan. M.P., F.G.S. and L.S. were supported from S grant of MEYS, and QK 1910156 project of National Agency for Agricultural Research, Czech Republic.

## Institutional Review Board Statement

The authors declare no experiments conducted on humans in the present study. The study was approved by the Institutional Review Board of Kazakh Research Institute of Livestock and Fodder Production (ТОО “КазНИИЖиК”), protocol code 0115РК02596, date of approval July 25, 2014.

## Data Availability Statement

The data presented in this study are available on request from the corresponding author.

## CRediT authorship contribution statement

**Nurlan Malmakov:** Conceptualization, Methodology, Investigation, Writing – original draft, Project administration, Resources. **Martin Ptacek:** Methodology, Formal analysis, Writing – review & editing, Project administration, Resources. **Filipp Georgijevic Savvulidi:** Methodology, Writing – review & editing. **Ludek Stadnik:** Methodology, Writing – review & editing, Supervision, Resources.

## Declaration of Competing Interest

The authors declare that they have no known competing financial interests or personal relationships that could have appeared to influence the work reported in this paper.
